# Editorial: Circuit, molecular, and developmental mechanisms in decision-making behavior

**DOI:** 10.3389/fnins.2023.1192237

**Published:** 2023-04-25

**Authors:** Tom Macpherson, Minae Niwa, Hirofumi Morishita, Takatoshi Hikida

**Affiliations:** ^1^Laboratory for Advanced Brain Functions, Institute for Protein Research, Osaka University, Osaka, Japan; ^2^Department of Psychiatry and Behavioral Neurobiology, Heersink School of Medicine, The University of Alabama at Birmingham, Birmingham, AL, United States; ^3^Department of Psychiatry, Icahn School of Medicine at Mount Sinai, New York, NY, United States

**Keywords:** cognition, decision-making, attention, cortical-basal ganglia circuits, behavior

Decision-making is a multifaceted cognitive process that forms a crucial aspect of our daily lives. As such, elucidating the cellular, molecular, and developmental mechanisms of decision-making is essential for gaining a comprehensive understanding of human behavior, as well as for developing new and effective treatments for decision-making impairments in clinical conditions. Here we summarize recent findings from multi-disciplinary and multi-species studies published in a recent Research Topic of Frontiers in Neuroscience, particularly focusing on how technological and methodological advancements have helped to illuminate the neural mechanisms underlying decision-making.

At the systems level, a series of parallelly organized cortico-basal ganglia-thalamo-cortical (CBGTC) loop circuits are known to play key roles in controlling decision-making and behavior by integrating sensory information necessary for action selection and generating appropriate motor responses based on the outcome of the decision-making process (Haber, 2003; Jin et al., 2014; Macpherson and Hikida, 2019; Macpherson et al., 2021). While many early studies demonstrating the importance of the CBGTC structures in decision-making utilized conventional interventions of neural signalizing, including neurotoxic lesions, pharmacological manipulation, and electrical stimulation, a major drawback of such approaches is that they often work indiscriminately on intermingled neural circuits. Over the last decade, this complication has largely been overcome by the emergence of cell-type- and pathway-specific genetically encoded tools, enabling unprecedented specificity in the visualization and manipulation of neural networks (Sjulson et al., 2015; Shen et al., 2022). Indeed, the recent application of pathway-specific techniques, including chemicogenetics and optogenetics, in non-human primates has provided fine-grained functional elucidation of cortical projection pathways (Oguchi and Sakagami), identifying dissociable roles for dorsolateral prefrontal cortex (PFC) projections to the dorsal striatum and medial dorsal thalamus in working memory and choice behavior, respectively (Oyama et al., 2021), and demonstrating the importance of the ventrolateral PFC-to-dorsal striatum pathway in inhibitory control and patience (Oguchi et al., 2021). Downstream of the cortex, in the striatum too, the use of pathway-specific expression of neurotransmission-blocking tetanus toxin has recently revealed the precise roles of nucleus accumbens dopamine D1 or D2 receptor-expressing medium spiny neuron (D1-/D2-MSN) output pathways in cognitive flexibility. In an attentional set-shifting task in mice, neurotransmission in NAc D2-MSNs was found to be necessary for the ability to switch between two learnt cue-outcome associations (reversal learning), but not for the ability to switch to a novel behavioral strategy (set-shifting), indicating a dissociation in the neural circuits controlling different aspects of adaptive decision-making (Macpherson et al.).

Another technological breakthrough that has recently advanced research into decision-making is the development of semi-automated touch-screen-based operant systems. These systems not only provide greater sophistication and versatility in the types of cognitive tasks that can be performed (allowing the use of image/video stimuli) as well as more natural response methods (touch rather than lever press), but also allow similar tasks to be performed in rodents, non-human primates, and humans, increasing the translatability of decision-making research (Horner et al., 2013; Mar et al., 2013). The utility of this apparatus is demonstrated in the current Research Topic, where 3 original research papers used touch-screen-based behavioral tasks to elucidate different aspects of decision-making. Dexter et al. described the optimization of trial unique non-match to location task in a touch-screen operant chamber in mice, allowing for more accurate assessment of working memory, a fundamental cognitive process for decision-making. The authors then employed their optimized paradigm to demonstrate that inactivation of the medial PFC or N-methyl-D-aspartate (NMDA) receptors is able to disrupt working memory. Similarly, Norman et al. used the touch-screen operant chambers to demonstrate that activity in the anterior cingulate area (ACA) projection to the visual cortex (VIS) is required for adjusting attention when task demands are high in a 5-choice serial reaction time task (5CSRTT), but not when task demands are low when only 2 choices are available (2CSRTT). Indeed, optogenetic inhibition of the ACA-VIS pathway immediately before cue presentation impaired performance in the 5CSRTT, but not the 2CSRTT. Finally, Aomine et al. used a touch-screen-based task to reveal that knockout of the Importin α3 gene in mice (KPNA3 KO) results in increased motivation to instrumentally respond for sucrose under a progressive ratio schedule of reinforcement. Using network and graph-theoretical analyses the authors then revealed KPNA3 KO to increase interregional functional connectivity in mice that underwent the progressive ratio task, suggesting an important role for KPNA3 in motivational control in decision-making.

Finally, computational modeling is increasingly becoming a crucial tool in decision-making research, providing a framework to understand the underlying cognitive processes that lead to decisions. This is exemplified by the recent introduction of a novel homeostatic reinforcement learning (HRL) model that provides a mechanistic explanation of sodium appetite (Uchida et al.). This HRL was not only able to successfully reproduce homeostatic-like behaviors, including approach and avoidance behaviors to sodium according to the individual's internal state, but importantly, was also able to reproduce the paradoxical observations of intragastric infusion tests [intragastric intake of sodium does not change the level of sodium intake (Lee et al., 2019)], which could not be explained by classic drive reduction models of sodium intake. These data indicate that decision-making concerning sodium appetite can be understood as a reinforcement learning process and highlight the merit of applying such computational techniques to the investigation of decision mechanisms.

In summary, over the last decade remarkable progress has been in elucidating both the neural circuits, as well as computational processes, underlying various cognitive processes that contribute to decision-making, including working memory, attentional control, and cognitive flexibility. In the years to come, further implementation of pathway-specific genetic engineering methods, advanced cognitive tasks, and computational techniques will undoubtedly foster new insights into the complex circuit, molecular, and developmental mechanisms underlying decision-making in experimental animals and humans.

## Author contributions

TM wrote the manuscript. MN, HM, and TH provided advice and editing. All authors read and approved the final manuscript.
